# Assessing the Impact of Climate Change on Early Childhood Caries Within the Framework of Sustainable Developmental Goal 13: A Scoping Review

**DOI:** 10.7759/cureus.71872

**Published:** 2024-10-19

**Authors:** Gauri Kalra, Tanu Nangia, Yaman Kumar

**Affiliations:** 1 Pediatric Dentistry, Manav Rachna Dental College, Faridabad, IND; 2 Dentistry, Maulana Azad Institute of Dental Sciences, Delhi, IND

**Keywords:** climate change, dental caries, early childhood caries, food insecurity, greenhouse effect, pediatric dentistry, sdg

## Abstract

Climate change, a significant factor in global health disparities, has been linked to numerous health issues, including oral health disorders such as dental caries and enamel hypoplasia. Sustainable Developmental Goal 13 (SDG 13) accentuates immediate action to battle climatic changes and their complications. This scoping review aimed to explore the existing evidence in the literature linking SDG 13 with Early Childhood Caries (ECC). The review was carried out following the PRISMA (Preferred Reporting Items for Systematic Reviews and Meta-Analyses) guidelines. A rigorous search was done during the month of May 2024 using databases PubMed, Web of Science, Scopus, and Google Scholar with search MeSH terms related to climate change, Early Childhood Caries (ECC), and Sustainable Developmental Goal (SDG). Publications or abstracts were included only in English from 2015 onwards, with no restrictions on the type of study. A summary of the eligible studies was compiled, highlighting the countries where the research was conducted, the type of study designs used, the region, study aims, and key findings.

Additionally, the study results were analyzed to determine whether SDG 13 was addressed. The initial search provided 74 articles, of which 51 were duplicates, yielding 23 for screening. After applying the eligibility criteria, seven studies were finally reviewed. Two of the seven studies included were from the African continent (Kenya and Nigeria), and one was a multi-centric study involving various continents, Africa (Nigeria & Kenya), Asia (Saudi Arabia, Indonesia), and America (Canada, Brazil). Two other studies were conducted in the UK, China, and India, while one was from the USA and focused on how climate change impacts dental caries. Only three studies were found to be addressing SDG 13. The review established a plausible link between ECC and climate change factors, addressing the need to incorporate sustainable developmental strategies and eco-friendly preventive measures in pediatric dentistry.

## Introduction and background

The 2030 agenda of Sustainable Development Goals (SDGs) has gained the attention of all the developed and developing nations to address many important issues such as improvement in general health and education, ending hunger and poverty, and controlling climate change. As a call for action, the United Nations in 2015 adopted seventeen goals to cater to the challenges posed to the planet and human life [1]. Due to global warming, changes in climatic conditions have been a considerable concern, affecting the general and oral health of the people. There have been deaths of about 1.3 million, and 4.4 billion people have been harmed due to alterations in climatic conditions [2].

Various consequences of climate change may include increased incidence of respiratory disorders such as Asthma and bronchitis due to greenhouse gaseous emissions [3, 4]. In turn, such emissions are proven to be responsible for the ozone layer depletion of the atmosphere, releasing Methane and Nitrous oxide gases into the air [5, 6]. Rising temperatures lead to arid or semi-arid weather conditions, increasing the alkalinity of underground water, which might be responsible for leaching out of fluorides and other minerals in the groundwater. Such disturbances interrupt nature's balance, thereby affecting the overall health and well-being of human beings [7].

As part of general health, oral health is affected by global warming and other climatic alterations. Studies have linked the increased prevalence of dental caries in early childhood to respiratory disorders and gas emissions due to climatic change [8, 9]. Early Childhood Caries (ECC) is an issue of concern affecting not only the teeth in children up to 6 years of age but also causing detrimental effects on mental, social, and functional well-being. Further, the release of high concentrations of fluorides in groundwater due to global warming might increase the uptake of fluoridated water, affecting the hydroxyapatite structure of teeth and thereby making them prone to dental caries during the first years of life. Thus, the indirect link of climate change with a higher risk of ECC projects the Sustainable Developmental Goal 13 (SDG goal 13) crucial in enhancing dental care in the pediatric population [9]. SDG 13 goal aims to combat the climatic change crisis. It primarily focuses on building resilience to withstand climate change-related hazards, formulating preventive strategies and policies, improving knowledge and awareness regarding climate change factors, its preventive and curative measures, and promoting effective steps to support developing countries in managing climatic alterations [10]. Thus, SDG 13 goal actions may be linked to ECC control, developing recent preventive policies according to the current scenario of caries affecting preschool children, and developing international guidelines by international stakeholders to support developing nations.

Hence, it becomes imperative to identify evidence of the relation between ECC and climatic change and its related factors. Also, documentation of actions taken under SDG 13 reducing climatic alterations and dental caries since the inception of SDGs is inevitable. This scoping review aims to assess the relationship between ECC and climatic change and its associated factors within the framework of SDG 13.

## Review

Material & methods

The current review explores the role of climatic changes in caries prevalence in children under 71 months of age, and the Sustainable Development Goal (SDG) focused on the same issue. The review followed the standard PRISMA (Preferred Reporting Items for Systematic Reviews and Meta-Analysis) guidelines [11]. The PICO framework for the research question- is as follows:

P (Population): Children under 71 months of age; I (Intervention/Exposure): Exposure to climate change and its associated factors (disaster management, conservation of natural resources, human security); C (Comparison) Children with limited exposure to climate change and other variables; O (Outcome): Incidence, prevalence, or severity of Early Childhood Caries (ECC). Additionally, the focus of available literature on Sustainable Development Goal 13 (SDG 13) was also evaluated. The formulated research question was - What is the evidence between climate change and its associated factors (disaster management, conservation of natural resources, and human security) and Early Childhood Caries (ECC)?

The authors initially searched during May 2024 with broader keywords: Early childhood caries, climate change, greenhouse effect, food security, and SDG 13. From 2015 to April 2024, relevant studies were searched for in databases such as PubMed, Scopus, and Web of Science. Grey Literature was searched on Google Scholar.

Search strategy

The rigorous search was performed on databases- PubMed, Scopus, Web of Science, and Google Scholar following the predefined inclusion criteria covering the last ten years. After conducting the search, titles and abstracts were screened independently by both the reviewers (GK) and (TN) according to the eligibility criteria. Selected articles were downloaded and screened by the same authors and then cross-verified with each other following the removal of duplicates. The third reviewer (YK) outside the institute resolved any uncertainty in manuscript selection. The articles were screened, and data was extracted under variables- name of authors, year, type of study, region, study aim, and main findings from the study. Additionally, study findings were correlated with an SDG13 indicator. MeSH (Medical Subject Headings) terms were used in combination with “AND” and “OR” to structure the search strategy as -

("Early childhood caries" OR "ECC" OR "dental caries" OR "tooth decay" OR "pediatric dental health" OR “dental cavities”) AND ("climate change" OR "global warming" OR "temperature change" OR "extreme weather" OR "climate variability") OR “Environmental Health” OR “ Food Security” OR “Greenhouse Effect” AND ("Sustainable Development Goals" OR "SDGs" OR "SDG 13" OR "climate action" OR "sustainability")

Eligibility Criteria

Inclusion criteria: Studies published in English were included in the review; however, studies in foreign languages with abstracts published in English were also considered to be included in the review. Publications (observational studies, randomized control trials, archaeological studies, editorials, opinions, short commentaries, case studies) except reviews (narrative, scoping, and systematic) were included in the study from 2015 till April 2024. Characteristics studied were the type of study, presence of aim and objective, conclusion and Inference, authors, year, region, and SDG principle being addressed or not.

Exclusion criteria: Review studies and studies not in English were excluded. Studies that exclusively addressed ECC or climate change were also excluded from the review.

Data Extraction and Synthesis

Following the inclusion and exclusion criteria, two reviewers (GK, TN) thoroughly selected the relevant studies and downloaded the abstracts for the selected studies. Additionally, a manual search strategy was followed to extract data unavailable in the initial database. Following this, full texts of potentially relevant manuscripts were retrieved and assessed independently by both researchers using the standardized format, and then the data was finally compiled in the excel sheet after verifying by the third author (YK).

Results

**Figure 1:**
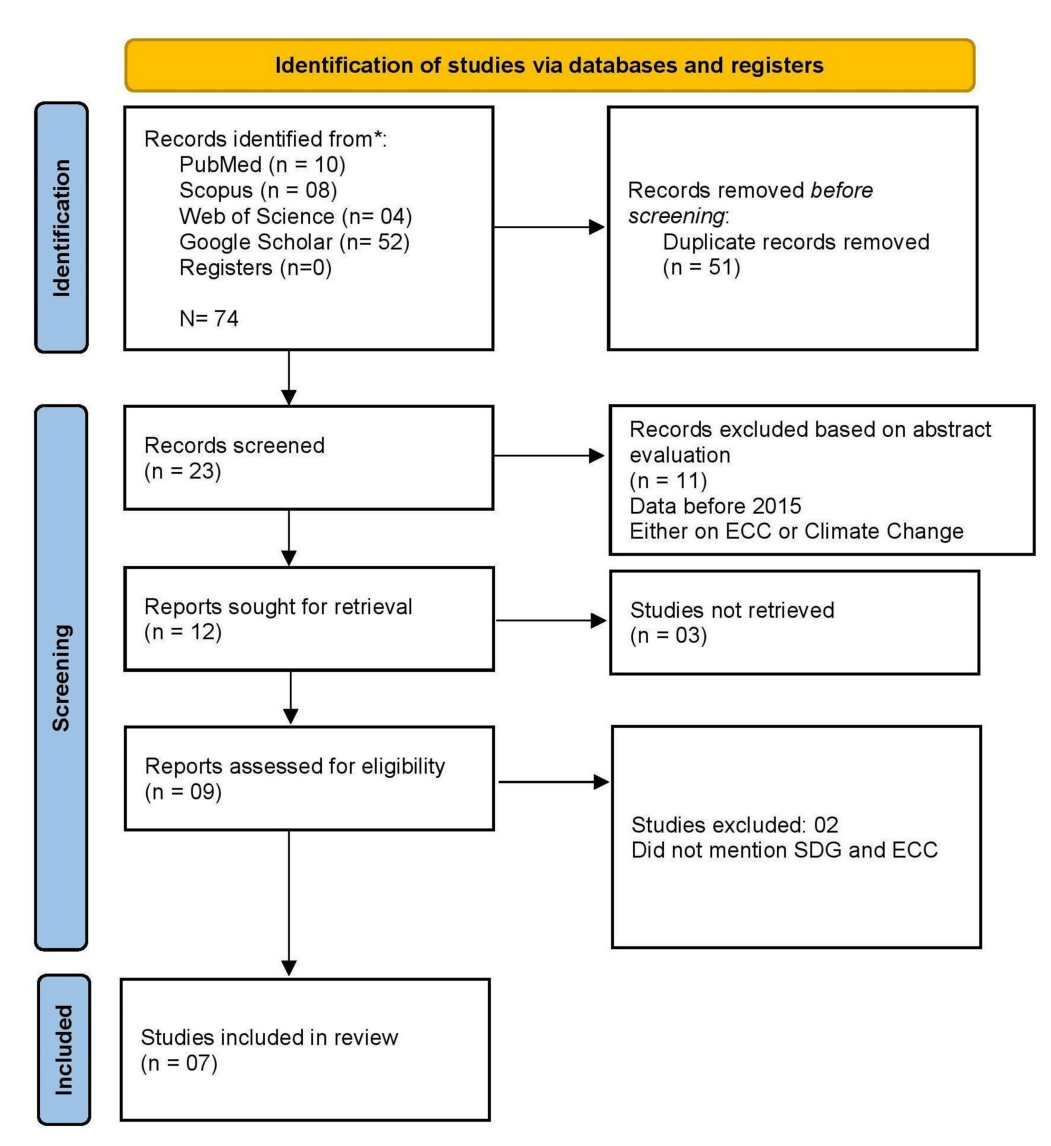
PRISMA Flow chart: Identification of studies through different databases PRISMA: Preferred Reporting Items for Systematic Reviews and Meta-Analysis

Following the PRISMA (Preferred Reporting Items for Systematic Reviews and Meta-Analysis) guidelines, after an initial electronic search, including 74 articles and the removal of duplicates, 23 articles were screened following the eligibility criteria by two independent reviewers (GK & TN). Employing the PICO framework, seven studies were identified at the end and extensively reviewed. (Figure1)

Characteristics of Studies

Out of the seven included studies, two papers were from the African continent (Kenya and Nigeria), one was a multicentric study from Africa (Nigeria, Kenya), Asia (Saudi Arabia, Indonesia), America (Canada, and Brazil), with two studies from the same primary author. One paper was from Europe (United Kingdom), and two from Asia (China and India) in 2015. One study from the USA discussed the various climatic factors adversely affecting dental caries. The study objectives were either directly or indirectly associated with the effects of climate change on the oral health of children, with the prevalence of ECC in the younger pediatric population, also highlighting the need for eco-friendly dental solutions for children to reduce the burden of dental caries and tackling the environmental challenges of oral health in the face of global climate disruption calls for thoughtful evaluation of interventions.

The study's findings mainly emphasized the link between climatic change, environmental sustainability, and dental health outcomes. These studies also described the application of detrimental dental materials that contribute to environmental degradation and climate change. Additionally, climatic shifts leading to changes in oxidative stress, heat emissions, and degrading air quality could further worsen oral health, thereby increasing dental sequelae such as dental caries.

The studies were also interrelated to the Sustainable Development Goal (SDG) 13, which focuses on climate action, including several targets to help fight climate change. Out of seven studies, about three were found to focus on SDG 13 goals, mainly targeting incorporating climate change actions into national policies, strategies, and planning. One study also focuses on applying caries prevention practices by observing an environmentally friendly approach. A detailed charting of the studies has been done and is described in Table 1.

**Table 1:** Summary of studies included in the scoping review

Author (Publication year)	Study site	Study design	Study objective	Findings	SDG mentioned
Kemoli AM, 2019 [12]	Kenya (Africa)	Editorial	Examining the influence of climate change on children's health, particularly oral health, and to identify the associated risks and challenges in pediatric dentistry as a result of environmental changes.	Global warming leads to increased temperatures, weather extremes, and environmental degradation, negatively impacting children's oral health. Rising carbon dioxide levels result in water acidification, affecting soil integrity, food sources, and increasing malnutrition, which in turn heightens susceptibility to dental issues like caries, periodontal disease, and dental erosion.	No
Cheung et al, 2019 [13]	China (Asia)	Archeological study (Non-dental)	Analysing the published stable isotope data and decayed teeth from remains of human skeletons at 77 archaeological areas in Chinese northern region.	The rapid alteration in the subsistence economy across northern and northwestern China was likely driven by climate change. This change prominently increased the prevalence of decayed teeth from before 4000 BP to after 4000 BP, mainly due to the increased consumption of cavity-causing starchy meals in the later period.	Yes, 13
Folayan MO et al, 2020 [8]	Nigeria (Africa)	Ecological study	Detecting the relation between 24 environmental indicators present globally and Early Childhood Caries in preschool children.	55.5% of preschoolers aged 3–5 years had Early Childhood Caries (ECC) among 61 countries. Two factors (Lower Methane Emission Intensity) and higher nitrous oxide emission intensity had significant associations with ECC prevalence.	No
Folayan MO et al, 2020 [6]	Nigeria, Egypt (Africa), Canada (Americas), Brazil (Europe), Indonesia, Saudi Arabia (Asia)	Ecological study	Determining the correlation between a level of environmental health, ecological vitality of the country, and prevalence of Early Childhood Caries (ECC) in young children.	ECC prevalence was inversely associated with both the Environmental Performance Index (EPI) and ecosystem vitality, though only the latter was significant for 3–5-year-olds. Conversely, a direct association was observed with environmental health.	No
Hackley DM et al (2021) [14]	USA (America)	Commentary/opinion	To broaden the discussion on the interruption of climatic conditions globally and include incorporation of dental health outcomes and preventive strategies for dental practice crisis.	Climate change is linked to a rise in temperature and worsening air quality. Both factors were recognized as risk factors for dental caries.	Yes, 13
Lyne A et al (2022) [15]	United Kingdom (Europe)	Intervention simulation study	Measuring the ecological effects of application of fluoride varnish among pediatric patients by comparative life cycle assessment method.	Flouride varnish application in children is beneficial most as a routine preventive protocol at individual private dental practices in comparison to school dental programs or children who do not visit any dental practice.	Yes, 13
Acharya S (2023) [16]	India (Asia)	Short Communication	To explore the impact of climate change on pediatric dentistry and identify environment-friendly solutions for sustainable dental practices.	The use of non- eco-friendly oral hygiene aids—such as toothbrushes and toothpaste made in plastic, non-biodegradable floss, plastic mouthwash packaging, and other patient utilities such as goody bags —along with one-time use plastics, considerably contributes to ecological harm, deteriorating community health, widening gaps in health, and increasing climate changes.	Yes, 13

Discussion

**Figure 2:**
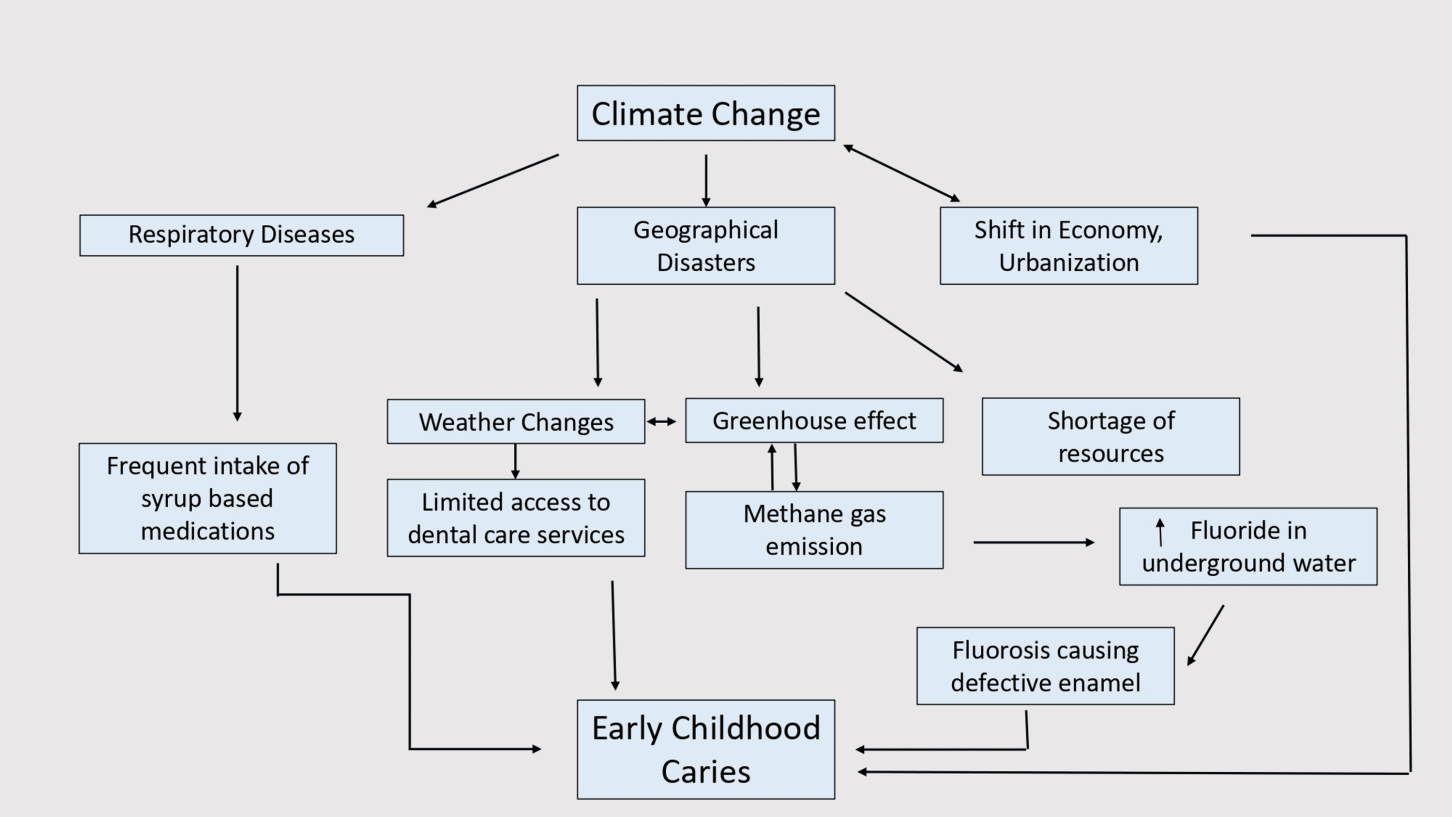
Relationship between climate change, its associated factors with Early Childhood Caries

Recently, for a decade, Climate change has been an issue of global concern as it has led to imbalances in the environmental ecosystem affecting all living beings. Human beings, too, have been affected by global warming issues, even though humanity has been primarily responsible for such dramatic changes on the earth. Oral and general health issues are prevalent due to climatic changes. The review highlights the lacuna of the literature on the correlation between climate change and ECC with additional information on whether the SDG 13 goal was addressed. Climate change, a massive global concern, has been found to affect the prevalence of caries either directly or indirectly. The cause-effect relationship between climate change and ECC is depicted in Figure 2.

The studies that were included in the review were found to have a significant relationship between climate change and dental caries. The studies first described how climatic changes have led to increased dental caries, including Early Childhood Caries (ECC) prevalence in preschool children [17]. This may be understood as worsening air quality, and air pollution has increased the prevalence and severity of respiratory diseases in every population [18]. Rising cases of Asthma and Chronic obstructive pulmonary disease (COPD) lead to the intake of heavy medications at a younger age, raising the occurrence of dental caries [19]. It is impossible to avoid contact between anti-asthmatic drugs and the oral cavity since the mode of administration is usually oral or through nebulizers. Medications being sugary in composition have been found to reduce the oral cavity's critical pH, rendering the tooth surface prone to caries [4]. Another concern is the increased greenhouse gaseous emissions, which increase the incidence of geophysical disasters and humanitarian crises, inhibiting access to dental care [20, 21, 22]. The inability to visit pediatric dentists or the unavailability of dental services, especially for children, are more likely to increase the risk of decay among younger children. Climatic alterations have also created arid conditions in some parts of the earth, leading to fluctuations in concentrations of minerals and levels of underground water table [7, 23]. As a result, concentration levels of minerals such as Fluorides have been found more in various parts of the world, leading to fluorosis and enamel hypoplasia and increasing caries prevalence [24]. Nonetheless, a weak association has been found between geothermal temperature variations and levels of fluoride in water [25].

Secondly, studies focused on the shift in the economy, industrialization, growth, and establishment of urban societies may have led to increased prevalence of ECC in children. Concurrent observations were reported by Tantawi ME et al. (2018), who studied statistics on caries in children in various countries under the United Nations and found higher ECC prevalence in highly industrialized nations [26]. Nevertheless, in the study by Folayan MO et al. (2020), besides ECC prevalence being correlated with urbanization, it was also stated that the presence of adequate ecosystem vitality, such as adopting healthy lifestyles and coherent use of resources, may offer protective effects against dental caries [6].

Thirdly, how the anticipatory and eco-friendly strategies of caries prevention can reduce harm to the environment and climate. The likelihood of a cause-effect relationship between dental caries prevalence and change in the climatic ecosystem has resulted in the incorporation of sustainability goals in pediatric dentistry. Thus, implementing eco-friendly measures in Pediatric dentistry may be achieved by applying fluorides and other non-invasive dental techniques, using recycled products, limiting plastics, amalgam-free dentistry, and proper waste disposal, which might make this field sustainable [16]. Consequently, correlating ECC and climate change with the Sustainable Development Goal (SDG 13) proved successful, as stated in our hypotheses. 

However, the authors could only find ecological studies or opinions and a life cycle assessment study being done to define the impact of climatic change in ECC or vice-versa. This might be because the plausible link between dental health and climatic conditions had been limitedly explored due to unawareness and limited knowledge. During our rigorous literature search, one recent scoping review conducted by Folayan et al. (2023) [9] focused on a similar topic, which included all types of studies with no time constraints. They reviewed six studies from 2007 to 2023; however, we included studies after 2015 to understand the 10-year trend of publications addressing the issue. Moreover, 2015 was dedicated to attaining the 2030 SDG agenda globally. The findings of the scoping review were concurrent with our study results, identifying data discussing how pediatric dental practices could contribute to climate change; in addition, we also reviewed the association of factors related to climate change causing ECC.

Futuristic Scope

Prospective research on evaluating the effects of climate change and ECC will help to enhance holistic health impact in children and aid in fabricating climate-resilient and sustainable health interventions aligned with the sustainability goals. Such opportunities encourage Interprofessional partnerships between climate scientists and dental experts, integrating eco-friendly solutions. Further, long-term research might also help track climatic change's influence on oral health over time. Accordingly, preventive policies, awareness initiatives, and innovative strategies may be implemented.

Limitations

To address the limitation of including literature available only in English, we minimized this by incorporating articles with abstracts available in English. This approach reduced the risk of excluding relevant manuscripts published in other languages. Additionally, the articles were limited to a specific age group (up to 71 months) as our research focused on evaluating the association between climate change and Early Childhood Caries.

## Conclusions

Early Childhood Caries has become increasingly prevalent among younger children, driven by multiple contributing factors. Recent reviews suggest a possible association between change in climate and dental health outcomes, including dental caries in children. Although current literature does not provide sufficient evidence to establish a definitive correlation, more evidence-based studies are needed to further explore the association between climate change and caries and its broader health impacts.

Such findings could be instrumental in shaping sustainability-focused policies aimed at controlling the prevalence of ECC and other oral health issues while mitigating the complex effects of climate change on ecosystems. In addition, guidelines promoting eco-friendly or green dentistry, particularly in pediatric care, should be endorsed by global dental health organizations. Developing specialized courses or curricula centered on sustainable dental practices would benefit communities worldwide.

## References

[1] (2024). Transforming our world: The 2030 agenda for sustainable development. https://sdgs.un.org/2030agenda.

[2] Wallemacq P, House R (2024). Economic Losses, Poverty and Disasters: 1998-2017. United Nations Office for Disaster Risk Reduction. https://www.undrr.org/publication/economic-losses-poverty-disasters-1998-2017.

[3] Samec T, Amaechi BT, Jan J (2021). Influence of childhood asthma on dental caries: A longitudinal study. Clin Exp Dent Res.

[4] Pacheco-Quito EM, Jaramillo J, Sarmiento-Ordoñez J, Cuenca-León K (2023). Drugs prescribed for asthma and their adverse effects on dental health. Dent J (Basel).

[5] Mar KA, Unger C, Walderdorff L, Butler T (2022). Beyond CO2 equivalence: The impacts of methane on climate, ecosystems, and health. Env Sci and Pol.

[6] Folayan MO, El Tantawi M, Schroth RJ, Kemoli AM, Gaffar B, Amalia R, Feldens CA (2020). Association between environmental Health, ecosystem vitality, and early childhood caries. Front Pediatr.

[7] Podgorski JE, Labhasetwar P, Saha D, Berg M (2018). Prediction modeling and mapping of groundwater fluoride contamination throughout India. Environ Sci Technol.

[8] Folayan MO, Tantawi ME, Gaffar B (2020). An ecological study of the association between environmental indicators and early childhood caries. BMC Res Notes.

[9] Foláyan MO, Schroth RJ, Abodunrin O (2024). Early childhood caries, climate change and the sustainable development goal 13: a scoping review. BMC Oral Health.

[10] (2024). Goal 13: Take urgent action to combat climate change and its impacts- United Nations Sustainable Development. https://www.un.org/sustainabledevelopment/climate-change/.

[11] Page MJ, McKenzie JE, Bossuyt PM (2021). The PRISMA 2020 statement: an updated guideline for reporting systematic reviews. BMJ.

[12] Kemoli AM (2019). Paediatric oral health and climate change. Edorium J Dent.

[13] Cheung C, Zhang H, Hepburn JC, Yang DY, Richards MP (2019). Stable isotope and dental caries data reveal abrupt changes in subsistence economy in ancient China in response to global climate change. PLoS One.

[14] Hackley DM (2021). Climate Change and Oral Health. Int Dent J.

[15] Lyne A, Ashley P, Johnstone M, Duane B (2022). The environmental impact of community caries prevention - part 1: fluoride varnish application. Br Dent J.

[16] Acharya S (2023). The impact of climate change on paediatric dentistry. Indian J Dent Res.

[17] (2024). Effects of climate change on health. Centers for disease control and prevention.

[18] Tran HM, Tsai FJ, Lee YL (2023). The impact of air pollution on respiratory diseases in an era of climate change: A review of the current evidence. Sci Total Environ.

[19] Arafa A, Aldahlawi S, Fathi A (2017). Assessment of the oral health status of asthmatic children. Eur J Dent.

[20] (2024). World Metrological Organization. Weather-related disasters increase over past 50 years, causing more damage but fewer deaths.. https://wmo.int/media/news/weather-related-disasters-increase-over-past-50-years-causing-more-damage-fewer-deaths.

[21] (2024). How climate change drives humanitarian crises. https://www.rescue.org/article/how-climate-change-drives-humanitarian-crises.

[22] Joury E (2019). Syria profile of the epidemiology and management of early childhood caries before and during the time of crisis. Front Public Health.

[23] Pörtner HO, Roberts DC, Adams H (2022). Climate change 2022: impacts, adaptation and vulnerability. IPCC. Nat Commun.

[24] Brahmbhatt SM, Rawat A, Sharma A, Urooge A, Pathak S, Bardhan D (2023). Enamel hypoplasia and dental fluorosis in children with special healthcare needs: an epidemiological study. Cureus.

[25] Podgorski J, Berg M (2022). Global analysis and prediction of fluoride in groundwater. Nat Commun.

[26] El Tantawi M, Folayan MO, Mehaina M (2018). Prevalence and data availability of early childhood caries in 193 United Nations Countries, 2007-2017. Am J Public Health.

